# Optimising Performance Through Sleep: An Evidence-Based Review

**DOI:** 10.7759/cureus.111990

**Published:** 2026-07-03

**Authors:** Daniel Bastock, Raj Amarnani

**Affiliations:** 1 Orthopaedics, Shrewsbury and Telford Hospital, Shrewsbury, GBR; 2 Internal Medicine, University College Hospital, London, London, GBR

**Keywords:** athletic therapy, sleep, sleep problems, sleep quality and quantity, sleep quantity

## Abstract

Sleep is increasingly being recognised as an important factor influencing athletic performance, recovery and overall health. This narrative review aims to discuss the factors that can affect sleep in athletes and how sleep can affect performance. Traditionally, athlete well-being has focused on training and nutrition; however, new evidence and initiatives now highlight sleep as an important element of performance. This review examines current evidence on sleep duration, sleep quality and sleep disturbances in athletic populations, with a particular focus on the effects related to competition. Athletes often report shorter sleep durations than non-athletes, despite experiencing less sleep fragmentation and quicker sleep onset. One study found that on nights before a competition day, athletes went to bed earlier (22:56 hr ± 49.2 min), woke up later (8:12 hr ± 58.6 min) and slept for longer (7:57 hr ± 60.9 min). A significant number of athletes face poor sleep quality, difficulty falling asleep, and excessive daytime sleepiness. Sleep disruptions are also common around competitions, with post-competition nights showing reductions in total sleep time and sleep quality compared to pre-competition nights. Factors contributing to this include late competition times, heightened physiological and psychological arousal, increased caffeine intake, travel demands and use of technology. Overall, these findings demonstrate that inadequate and disturbed sleep is widespread among athletes, especially during competition periods, which may impact recovery, cognitive function and emotional stability.

## Editorial

Introduction

In recent years, there has been increasing recognition of the importance of sleep for athletic performance and overall athlete health. Historically, diet and exercise were regarded as the primary determinants of athlete well-being and consequently received the majority of research attention. [1] However, in 2022, the International Olympic Committee (IOC) formally acknowledged sleep as a key contributor to athlete function and performance, introducing the online platform Sleep to Compete [2]. This evolving perspective has led numerous sporting organisations to incorporate dedicated sleep coaches into their athlete support teams. We narrowed the search to articles on PubMed, with the search criteria involving the keywords 'sleep', 'athletes' and 'performance' from 2019 onwards. We then chose four peer-reviewed articles that were accessible in the public domain at the time of creation of the narrative. Based on these criteria, there was a gap in the knowledge of what factors can affect sleep and how sleep can affect performance in one article.

Sleep disorder prevalence in athletes

Insufficient sleep in athletes has been well documented, with the average hours of sleep being 6.5 ± 0.43 hours across sports, compared to 7.11 ± 0.25 hours in non-athletes [1,3]. Despite this, athletes had less sleep fragmentation and lower sleep onset latency. [1] Multiple studies have shown that elite athletes have poorer sleep quality than non-athletes, with 21.8% of athletes having difficulty falling asleep at least three times a week, measured by the Pittsburgh Sleep Quality Index [4]. The same study then showed that 51% of student athletes reported a score equal to or greater than 10 on the Epworth Sleepiness Scale, indicating that student athletes are sleepy during the day [4,5]. This may be a reason for this difference; however, other reasons may be due to differences in caffeine intake, training schedules and time zone traveling for competitions. [3] The use of smartphones may also disrupt sleep, with athletes posting on social media usually after training and matches [3]. Further, blue light emitted from phone screens can disrupt melatonin production, leading to inadequate sleep and morning sleepiness [3].

Sleep before and after a competition

There are sleep quality and length differences between nights before and after competitions for elite athletes. One study found that on nights before a competition day, athletes went to bed earlier (22:56 hr ± 49.2 min) (5), woke up later (8:12 hr ± 58.6 min) and slept for longer (7:57 hr ± 60.9 min). On nights after competition, sleep was negatively impacted across nearly all variables (onset: 25:09 hr ± 129.9 min; offset: 8:05 hr ± 88.2 min; and total sleep time (TST): 5:51 hr ± 107.1 min). Finally, caffeine consumption was significantly higher based on servings (2.15 ± 0.82) on competition days compared to rest days (1.95 ± 0.71) and training days (2.02 ± 0.70) [5].

There may be numerous reasons for this discrepancy, with one of the key factors being the late evening/nighttime competition timings. This can cause increased adrenaline and cortisol production, as well as muscle soreness and pain, leading to decreased sleep quality [5]. The psychological aspect of performing later in the day and then thinking about performance and how they could have improved can also play a role in sleep quality [5,1].

The data for the pre-competition sleep might be attributed to the athlete's own awareness and knowledge of the importance of sleeping well before a competition, meaning they may sleep earlier and engage in better sleep hygiene habits [5].

Post-competition sleep is typically poorer than pre-competition sleep, as previously discussed. One contributing factor may be increased caffeine consumption, often used by athletes to enhance alertness and performance - particularly when competitions are scheduled close to their usual bedtime [3]. This stimulant effect, combined with the physiological and psychological arousal following competition, likely disrupts sleep quality and duration [5]. Another contributing factor may be the increased alcohol consumption post-competition, either for celebration or as part of team bonding, such as a post-match meal. The alcohol reduces rapid eye movement sleep duration and increases sleep disturbances [5]. However, this may not be the case for all athletes, as some may be under the legal age for alcohol consumption or be observing religious beliefs that forbid alcohol.

Conclusion

With growing research highlighting the important role of sleep in athletic performance and recovery, sleep is now being recognised as a pillar of athlete health, alongside training and nutrition. The evidence suggests that both the quality and quantity of sleep can be compromised in elite athletes, particularly around competition periods. This not only impacts physical recovery but also cognitive and emotional readiness. As such, optimising sleep should be a key part of any performance strategy. Future work should continue to explore tailored interventions, including sleep hygiene education, scheduling adjustments and potential support from sleep coaches, to help athletes recover more effectively and perform at their peak.
